# Polymorphic variants in Sweet and Umami taste receptor genes and birthweight

**DOI:** 10.1038/s41598-021-84491-4

**Published:** 2021-03-02

**Authors:** Riccardo Farinella, Ilaria Erbi, Alice Bedini, Sara Donato, Manuel Gentiluomo, Claudia Angelucci, Antonella Lupetti, Armando Cuttano, Francesca Moscuzza, Cristina Tuoni, Cosmeri Rizzato, Massimiliano Ciantelli, Daniele Campa

**Affiliations:** 1 Department of Biology, University of Pisa, Pisa, Italy; 2 Division of Neonatology, Santa Chiara Hospital, Via Roma, 67, 56126 Pisa, Italy; 3 Department of Translation Research and of New Technologies in Medicine and Surgery, University of Pisa, Pisa, Italy

**Keywords:** Paediatric research, Genetic association study

## Abstract

The first thousand days of life from conception have a significant impact on the health status with short, and long-term effects. Among several anthropometric and maternal lifestyle parameters birth weight plays a crucial role on the growth and neurological development of infants. Recent genome wide association studies (GWAS) have demonstrated a robust foetal and maternal genetic background of birth weight, however only a small proportion of the genetic hereditability has been already identified. Considering the extensive number of phenotypes on which they are involved, we focused on identifying the possible effect of genetic variants belonging to taste receptor genes and birthweight. In the human genome there are two taste receptors family the bitter receptors (TAS2Rs) and the sweet and umami receptors (TAS1Rs). In particular sweet perception is due to a heterodimeric receptor encoded by the *TAS1R2* and the *TAS1R3* gene, while the umami taste receptor is encoded by the *TAS1R1* and the *TAS1R3* genes. We observed that carriers of the T allele of the *TAS1R1*-rs4908932 SNPs showed an increase in birthweight compared to GG homozygotes Coeff: 87.40 (35.13–139.68) p-value = 0.001. The association remained significant after correction for multiple testing. *TAS1R1*-rs4908932 is a potentially functional SNP and is in linkage disequilibrium with another polymorphism that has been associated with BMI in adults showing the importance of this variant from the early stages of conception through all the adult life.

## Introduction

The first thousand days of life from conception have a significant impact on the health status with short and long-term effects for each individual^1^. Among several anthropometric and maternal lifestyle parameters birth weight plays a crucial role on the growth and neurological development of infants^2^. For example, SGA infants (small for gestational age: birth weight < 3rd percentile for their gestational age) have been reported to show several problems that could lead to increased morbidity and mortality in the perinatal period^3^. In addition, many studies show that both SGA infants as well as LGA infants (large for gestational age: birth weight > 97th percentile for their gestational age) have an increased risk to develop, at later ages of their life, several health conditions, such as, cardiovascular diseases^4^, obesity^5^, type 2 diabetes^6^, arterial hypertension^7^ and chronic renal insufficiency^8^. In addition, children and adolescents delivered SGA have often highlighted cognitive and relational disorders, communication and concentration difficulties and therefore poor academic performance^9^.

Foetal growth, on which birth weight depends, is the result of the interaction of numerous and complex genetic, epidemiologic and environmental variables. Recent genome wide association studies (GWAS) have demonstrated a robust foetal and maternal genetic background of birth weight, however only a small proportion of the genetic hereditability has been already identified^10–13^ and the discovery of novel loci is warranted. Several of the single nucleotide polymorphisms (SNPs) associated with birth weight have been suggested to play a role in determination of glycaemic traits in adults^14^, suggesting a link between the genetics of the metabolism regulation in the newborns and in adults. Considering all these premises, we focused on identifying the possible effect of genetic variants belonging to taste receptor genes and birthweight. In the human genome there are two taste receptors family the bitter receptors (TAS2Rs) and the sweet and umami receptors (TAS1Rs). In particular, sweet perception is due to a heterodimeric receptor encoded by the *TAS1R2* and the *TAS1R3* gene while the umami taste receptor is encoded by the *TAS1R1* and the *TAS1R3* genes.

TAS gene expression has been firstly identified in the tongue, but subsequently in a multitude of human tissues and organs. TAS genes have various functions, alongside tasting, such as gut motility, glucose homeostasis, defense against bacterial infection in the upper airway tract^15–20^. SNPs belonging to these genes have been thoroughly studied in relation to a multitude of human traits, among which nicotine dependence, caloric intake, obesity, body mass index (BMI), food and beverage acceptance, ageing and various human neoplasms^21–31^.

Polymorphisms in the *TAS1R*s family have been associated to food intake and overweight in children^32^ and adults^33^, sweet taste and sucrose detection threshold and sensitivity^34,35^ and with food intake and gastric cancer^36^. We investigated the effect of polymorphic variants in the *TAS1R1*, *TAS1R2* and *TAS1R3* genes on birth weight on 1077 newborns of Caucasian origin.

## Results

Among the 1077 full term-newborns recruited, 25 (2.3%) were discarded because they had a genotyping call rate lower than 75%. The average SNP call rate of the remaining samples was 98.70%, with a minimum of 95.64% for *TAS1R3-*rs111615792 and a maximum of 99.53% for *TAS1R1-*rs17029626 and for *TAS1R1-*rs4908563. The QC analysis showed a concordance among duplicates greater than 99%. All the SNPs allelic and genotyping frequencies resulted in Hardy–Weinberg equilibrium (p-value > 0.01). The average weight measured for males was 3376 g and 3251 g for females. The average gestational time was 39 + 4 weeks (39 + 3 for males and 39 + 4 for females). The relevant characteristics of the population are given in Table 1.

**Table 1:** Characteristics of the population in study.

Variable	Males (n = 554)	Females (n = 495)	Total
Average gestational age^a^	39 + 3	39 + 4	39 + 4
Average birthweight^b^	3375.620	3251.116	3316.896
Average length^c^	50.648	50.120	50.392
Average head circumference^c^	34.940	34.226	34.605
Mother's age^d^	33 years and 321 days	34 years and 77 days	34 years and 14 days
Pre-gravidic BMI^e^	22.458	23.162	22.866
Weight increase during pregnancy^f^	13,346	12,811	13,057
Smoking status of the mother (yes/no)^g^	27/522	21/467	48/989
Maternal gestational diabetes (yes/no)^h^	83/467	78/415	161/882
Maternal pre-gravidic diabetes (yes/no)^b^	7/543	5/486	12/1029

^a^This information was available for 1046 subjects. The measurement unit is “weeks”.

^b^This information was available for 1041 subjects. The measurement unit is “g”.

^c^This information was available for 733 subjects. The unit of measure is “cm”.

^d^This information was available for 1029 subjects. The unit of measure is “years”.

^e^This information was available for 731 subjects. The measurement unit is “kg/m^2^”.

^f^This information was available for 736 subjects. The unit of measure is “g.

^g^This information was available for 1037 subjects; h-This information was available for 1043 subjects.

### Results of the association analysis between epidemiologic variables and genotypes on birthweight

We performed a linear regression analysis to evaluate the association with the genetic and non-genetic variables and birthweight. We observed that birthweight is significantly associated with sex with males been heavier than females, Coeff = 124.5; 95% CI (72.17–176.83); p-value = 3.11 × 10^–6^. Gestational age showed a very strong proportional effect in birthweight increase with longer gestational time (expressed in weeks) Coeff = 160.77; 95% CI (140.96–180.58); p-value = 5.72 × 10^–57^. We also observed an inverse association between maternal smoking and birthweight with a coeff = − 202.95; 95% CI (− 328.09 to 77.81); p-value = 0.001). The results are shown in Table 2.

**Table 2:** Results of the association analysis between clinical and anthropometric variables and birthweight.

Covariates	Coeff. (CI)	p-value
Sex^a^	124.50 (72.17 to 176.83)	3.11 × 10^–06^
Gestational age^b^	160.77 (140.96 to 180.58)	5.72 × 10^–57^
Maternal age^c^	− 4.37 (− 9.40 to 0.66)	0.088
Maternal gestational diabetes^d^	17.11 (− 56.11 to 90.33)	0.647
Maternal pregravidic diabetes^e^	18.34 (− 228.96 to 265.65)	0.884
Maternal smoking behavior^a^	− 202.95 (− 328.09 to 77.81)	0.001
Pre-gravidic BMI^g^	4.45 (− 2.20 to 11.09)	0.190
Weight increase during pregnancy^h^	12.52 (5.78 to 19.26)	2.5 × 10^–04^

^a^This information was available for 1037 subjects.

^b^This information was available for 1046 subjects.

^c^This information was available for 1029 subjects.

^d^This information was available for 1043 subjects.

^e^This information was available for 1041 subjects.

^f^This information was available for 733 subjects.

^g^This information was available for 731 subjects.

^h^This information was available for 726 subjects.

All the analysis conducted to establish the effect of genetic variables were corrected for these three variables. We observed four associations between the genotypes and birthweight, however only one resulted statistically significant after correction for multiple testing. In specific, heterozygous carriers of the T allele of the *TAS1R1*-rs4908932 SNP showed an increase in birthweight compared to GG homozygotes Coeff: 87.40 (35.13–139.68) p-value = 0.001. We observed an additional association in the *TAS1R1* gene and birthweight, namely the positive effect of the G allele of *TAS1R1*-rs4908930 Coeff_rs4908930_ = 50.60; 95% CI (0.70–100.49); p-value = 0.047, compared to the common A homozygotes. Finally, we observed two associations in the *TAS1R2* gene, an increase in birthweight for the G allele homozygotes of *TAS1R2*-rs4920566 SNP (Coeff = 71.65; 95% CI (2.40–140.91); p-value = 0.043) and for the carriers of the G allele of the TAS1R2-rs9701796 (Coeff = 136.98; 95% CI (7.80–266.16); p-value = 0.038). The SNPs selected for the *TAS1R3* gene did not show any statistically significant association with birth weight. The results of these analysis are shown in Table 3.

**Table 3:** Results of the association analysis between epidemiologic variables and genotypes on birthweight**.**

GENE_SNP	Alleles	Genotypes			Cod-het		Cod-rec	
		MM	Mm	mm	Coeff (IC)	p-value	Coeff (IC)	p-value
*TAS1R1_rs11587438*	T/C	713	254	27	− 23.35 (− 78.06 to 31.36)	0.403	96.35 (− 50.50 to 243.20)	0.198
*TAS1R1_rs12080675*	A/C	686	295	41	1.63 (− 50.48 to 53.74)	0.951	72.35 (− 48.04 to 192.74)	0.239
*TAS1R1_rs12132145*	G/A	427	469	127	− 20.98 (− 70.93 to 28.97)	0.410	− 35.04 (− 110.56 to 40.49)	0.363
*TAS1R1_rs12565181*	G/A	760	235	25	18.76 (− 36.97 to 74.50)	0.509	− 7.48 (− 159.20 to 144.24)	0.923
*TAS1R1_rs17029626*	A/G	826	189	12	27.68 (− 32.64 to 88.00)	0.368	31.95 (− 185.93 to 249.82)	0.774
*TAS1R1_rs4908563*	T/C	307	527	192	14.67 (− 39.15 to 68.49)	0.593	3.92 (− 65.03 to 72.86)	0.911
*TAS1R1_rs4908930*	A/G	459	443	116	50.60 (0.70 to 100.49)	**0.047**	28.17 (− 49.71 to 106.04)	0.478
*TAS1R1_rs4908932*	G/T	717	283	27	87.40 (35.13 to 139.68)	**0.001**	25.20 (− 120.80 to 171.20)	0.735
*TAS1R2_rs12028479*	G/T	864	149	7	− 8.30 (− 74.61 to 58.00)	0.806	− 148.49 (− 431.57 to 134.60)	0.304
*TAS1R2_rs12033832*	G/A	473	436	84	− 19.87 (− 69.67 to 29.94)	0.434	− 55.85 (− 144.81 to 33.11)	0.219
*TAS1R2_rs12137730*	A/C	436	460	120	17.41 (− 32.63 to 67.46)	0.495	19.08 (− 58.14 to 96.29)	0.628
*TAS1R2_rs3935570*	G/T	494	457	74	− 30.53 (− 79.12 to 18.06)	0.218	− 29.43 (− 122.96 to 64.11)	0.538
*TAS1R2_rs4920564*	T/G	380	504	133	− 6.19 (− 57.13 to 44.75)	0.812	− 35.86 (− 111.48 to 39.76)	0.353
*TAS1R2_rs4920566*	A/G	401	456	164	20.73 (− 30.44 to 71.90)	0.427	71.65 (2.40 to 140.91)	**0.043**
*TAS1R2_rs6662276*	C/T	918	98	1	4.82 (− 75.02 to 84.66)	0.906	188.75 (− 562.65 to 940.16)	0.622
*TAS1R2_rs9701796*	C/G	688	293	35	− 49.43 (− 101.44 to 2.57)	0.062	136.98 (7.80 to 266.16)	**0.038**
*TAS1R3_rs111615792*	G/A	949	37	0	− 74.51 (− 200.38 to 51.36)	0.246	n.c	−
*TAS1R3_rs11488701*	C/T	959	61	3	31.15 (− 67.88 to 130.17)	0.538	− 83.52 (− 516.78 to 349.73)	0.706
*TAS1R3_rs139522421*	C/A	953	65	3	5.18 (− 90.98 to 101.35)	0.916	− 111.67 (− 546.19 to 322.85)	0.614
*TAS1R3_rs307374*	C/T	971	54	1	40.88 (− 63.12 to 144.88)	0.441	304.85 (− 439.22 to 1048.93)	0.422
*TAS1R3_rs3813210*	C/T	915	100	5	9.85 (− 69.06 to 88.76)	0.807	− 215.18 (− 551.02 to 120.66)	0.209

Values in bold are significant at the conventional level of p < 0.05.

### Functional relevance of the SNPs

In the GTEx database there are no eQTLs for *TAS1R2*-rs9701796 and *TAS1R2*-rs4920566, while for *TAS1R1*-rs4908932 there is only one eQTL in the aorta artery. The database shows ten eQTLs for *TAS1R1*-rs4908930, but none in the gastro-enteric tract. RegulomeDB assigns a rank of 4 to *TAS1R1*-rs4908932 and *TAS1R1*-rs4908930 and a rank of 5 for *TAS1R2*-rs9701796 and *TAS1R2*-rs4920566. Haploreg, shows that all the SNPs are situated in potentially methylated regions and that they alter a potential TF binding site, although with a null or modest effect.

## Discussion

Birthweight can have a dramatic impact in the development and health of the newborn in the short- period and in the long-term period. There are several well-known factors that have a strong effect on birthweight and genetic variability has been investigated in this regard. GWAS have identified a relatively small number of risk loci and the hereditability explained is still limited. We have taken into consideration the genetic variability in taste receptors since it has been repeatedly associated with BMI and metabolic traits, dietary behaviours and human pathologies^15–31^. This study has been conducted on more than 1000 new-borns of Italian origin collected by the Santa Chiara Hospital of Pisa with the aim to further improve our knowledge on the epidemiologic and genetic factors that influence birthweight.

We observed a very strong effect of sex, gestational age and maternal smoking on the birthweight as expected and reported in the literature^37,38^.

The most novel findings of this study are represented by several associations between the SNPs and birthweight, in particular, we found two hits in the *TAS1R1* (rs4908930, rs4908932) and *TAS1R2* (rs4920566, rs9701796) genes. None of these SNPs has been previously reported with body weight in adults or children. The best finding, both considering the strength of the association (i.e. coefficient) and the statistical significance (p value) that we observed was the average increase of 87 g for the carriers of the T allele of *TAS1R1*-rs4908932 (p = 0.001). This finding is the only one that remains significant after multiple testing correction according to Bonferroni.

According to Haploreg *TAS1R1*-rs4908932 is situated 29 bp in the 3′-UTR of the *TAS1R1* gene and could modify the methylation status of the gene in 24 tissues among which several belonging to the GI tract including the colon, the liver, the stomach and the pancreas. It is interesting to note that the SNP possibly modifies the methylation status also in several foetal organs, including the intestine. In addition, Haploreg also suggests that the SNP could modify the binding of several transcription factors including HNF4A which is involved in the development of the intestines and mutation of which have been associated with metabolic diseases^39–42^. These indications suggest a potential key role of the SNP in regulating the gene expression that could translate in the differential birthweight observed. *TAS1R1*-rs4908932 is in moderate LD with several SNPs that have been found to be associated with human traits. Of particular relevance is rs6577584 (r^2^ = 0.114, D’ = 0.9402 in the European individuals of the 1000G project, according to LDlink) since it has been found to be associated, at genome wide level, with BMI in adults in a study conducted using the UK biobank repository^43^, highlighting the importance of the locus from foetal life to adulthood.

In addition to BMI variants in LD with *TAS1R1*-rs4908932 have also been reported to be associated with cardiovascular disease, age at menopause, and medication use as reported in the GWAS catalogue^43^, making this a potential pleiotropic locus of the genome.

The effect size on birthweight associated *TAS1R1*-rs4908932 observed in our study is rather large (87 g) compared with what reported by others^10,12,44^ for several SNPs associated with birthweight. For example, Beaumont and colleagues, in a study that included UK biobank data, reported a maximum effect size of around 50 g^10^. Even though, the SNPs are not the same and therefore not directly comparable, this difference could be at least partially explained by the size of the two studies with ours being smaller. It would be therefore important to replicate our finding in a larger cohort of individuals to better compute the effect of the variant.

A clear strength of this study is the fact that the individuals collected are consecutive and therefore they are an unbiased representation of the individuals born in a high-volume centre such as the Santa Chiara university hospital in Pisa. Moreover, population stratification is not an issue considering that all the subjects of the study have been collected in the same center.

In conclusion the association that we propose here between *TAS1R1*-rs4908932 and birthweight is corroborated by a study-wise significance and reflects the association of a locus on chromosome 1 that has been already observed in adults showing the importance of this variant from the early stages of conception through all the adult life.

## Materials and methods

### Study population

This study was carried out on 1077 new-born recruited at the Division of Neonatology of the Santa Chiara Hospital (Pisa, Italy) from 2015 to 2019. Inclusion criteria in the recruitment were term birth defined as a gestational age ≥ 37 weeks and five minutes Apgar score ≥ 7 that indicates that the newborn is in good health and does not require additional interventions or treatments^45,46^. Exclusion criteria consisted of an Apgar score < 7, suspicion of genetic syndrome or metabolic diseases. For each individual 5 ml of blood were collected from the cord at birth and anthropometric measures at birth (birth weight, length, head circumference) were retrospectively collected. In addition, mother's age, maternal smoking behavior, pre-pregnancy BMI, maternal gestational diabetes, maternal pre-gravidic diabetes, maternal weight increase during pregnancy were also collected. The parents of all subjects signed a written informed consent form and the study was approved by the ethical committee of the Meyer Children Hospital of Florence which is the appointed IRB for all the pediatric studies in the Tuscany region, all methods were performed in accordance with the relevant guidelines and regulations. Additional information has been given elsewhere^47^.

### Selection of polymorphisms

The selection of polymorphisms included in the study focused on tagging (tSNP) and functional SNPs. The choice of the tSNPs was based on the Linkage Disequilibrium (LD) in the Caucasian population. To select the tSNP of *TAS1R1, TAS1R2 and TAS1R3,* the chromosomal position of these genes was inserted in Ensemble genome browser's VCF to PED conversion tool (online version http://grch37.ensembl.org/Homo_sapiens/Tools/VcftoPed), which returns a linkage pedigree file and a marker information file with European population genotype data. These files were uploaded to Haploview, a bioinformatic software (https://www.broadinstitute.org/haploview/haploview version 4.2) used to choose tagging SNPs^48^. The search criteria for tSNP set on the Haploview software were: r^2^ > 0.8 and minimum allele frequency (MAF) > 0.05.

The choice of functional polymorphisms was focused on expression Quantitative Trait Loci (eQTLs), genetic variants, including SNPs, that can modify and regulate gene expression. The expression Single Nucleotide Polymorphisms (eSNPs) selected for this study are significant eQTL of the digestive tract, in particular at the level of the pancreas, liver, terminal ileum, transverse colon and sigmoid colon. The choice fell on these five tissues in view of a possible association with different perception and/or metabolization of nutrients with birthweight. The final list consisted in 21 SNPs among which 4 eQTLs.

### Samples preparation and genotyping

DNA was extracted using Quick-DNA Universal Kit (Zymo Research, Irvine, CA, USA) from cord blood that was collected during delivery and then stored frozen at – 20 °C. Genotyping was conducted in 384 well plates using TaqMan assays (Thermo Fisher Scientific, Waltham, Massachusetts, USA) as recommended by the provider. For quality control of the genotyping 36 (3.3%) duplicated samples were added and processed as the rest. Genotyping call was done using the QuantStudio 5 Real-Time PCR System (Applied Biosystems) and the QuantStudio Design & Analysis Software V1.4.3 (Thermo Fisher Scientific, Waltham, MA, USA).

### Statistical analysis

For each SNP Hardy–Weinberg equilibrium was assessed using Pearson chi-square test. To estimate how the anthropometric and epidemiologic variables and genetic variability affects birth weight a linear regression model was used, calculating the regression coefficient, its confidence interval (95% CI) and the p-value. If the regression coefficient takes positive values, it indicates that the average weight increases for carriers of the rare allele, on the contrary, in the case of negative values, the regression coefficient represents how much the birth weight is reduced on average in the presence of rare allele. For the genetic variables we used a codominant model of inheritance putting the most common allele as reference category in each analysis. Applying the Bonferroni correction (dividing the threshold value of 0.05 by the number of SNPs) we considered the p-value = 0.0024 as threshold for statistical significance.

### Bioinformatic tools

To test the functional relevance of the SNPs associated with birthweight we used three bioinformatic tools. The Genotype-Tissue Expression (GTEx- version v7release, GRCh37/hg19 assembly, data access on 04/01/2019) project to identify if the variants where associated with gene expression. Such variants are called expression quantitative trait loci (eQTLs). RegulomeDB 2.0 (https://www.regulomedb.org/regulome-search/) was used, instead, to assess whether the variant is associated with regulatory potential. RegulomeDB assigns to each SNP a rank, going from 1a to 7 in descending order of the accumulated evidences of functional relevance of the SNP. The accumulated evidence consists in several items such as whether the SNPs binds to one or more transcription factor, if it lies in a DNAse sensitive region (i.e. a region that is accessible to transcription factors), and it is an eQTL. Finally, we used Haploreg v4.1 (https://pubs.broadinstitute.org/mammals/haploreg/haploreg.php) to assess whether the variants belong to transcription factor binding sites or if they are in methylated DNA regions.

## Data Availability

The data for this work will be made available to researchers who submit a reasonable and detailed request to the corresponding author, conditional to approval of the Ethics Commission of the Meyer Children Hospital of Florence which is the appointed IRB for all the pediatric study in the Tuscany region. Data will be stripped from all information allowing identification of study participants.
